# Evaluation of Cutting-Tool Coating on the Surface Roughness and Hole Dimensional Tolerances during Drilling of Al6061-T651 Alloy

**DOI:** 10.3390/ma14071783

**Published:** 2021-04-04

**Authors:** Hamza A. Al-Tameemi, Thamir Al-Dulaimi, Michael Oluwatobiloba Awe, Shubham Sharma, Danil Yurievich Pimenov, Ugur Koklu, Khaled Giasin

**Affiliations:** 1 Mechanical Engineering Department, University of Baghdad, Baghdad Al-Jadriya 10070, Iraq; Hamza.al-tameemi@coeng.uobaghdad.edu.iq (H.A.A.-T.); dr.thamir72@coeng.uobaghdad.edu.iq (T.A.-D.); 2 22a, Guinness Road, Ogba/Ikeja, Lagos, Nigeria; michael.a@tranos.ng; 3 Department of Mechanical Eng., IKG Punjab Technical University, Jalandhar-Kapurthala Road, Kapurthala, Punjab 144603, India; shubham543sharma@gmail.com; 4 Department of Automated Mechanical Engineering, South Ural State University, Lenin Prosp. 76, 454080 Chelyabinsk, Russia; danil_u@rambler.ru; 5 Department of Mechanical Engineering, Faculty of Engineering, Karamanoglu Mehmetbey University, 70100 Karaman, Turkey; ugurkoklu@gmail.com; 6 School of Mechanical and Design Engineering, University of Portsmouth, Portsmouth PO1 3DJ, UK

**Keywords:** Al6061-T651, coating, TiAlN, TiN, TiN/TiAlN, drilling, surface roughness, hole size, circularity, perpendicularity, cylindricity

## Abstract

Aluminum alloys are soft and have low melting temperatures; therefore, machining them often results in cut material fusing to the cutting tool due to heat and friction, and thus lowering the hole quality. A good practice is to use coated cutting tools to overcome such issues and maintain good hole quality. Therefore, the current study investigates the effect of cutting parameters (spindle speed and feed rate) and three types of cutting-tool coating (TiN/TiAlN, TiAlN, and TiN) on the surface finish, form, and dimensional tolerances of holes drilled in Al6061-T651 alloy. The study employed statistical design of experiments and ANOVA (analysis of variance) to evaluate the contribution of each of the input parameters on the measured hole-quality outputs (surface-roughness metrics *R*_a_ and *R*_z_, hole size, circularity, perpendicularity, and cylindricity). The highest surface roughness occurred when using TiN-coated tools. All holes in this study were oversized regardless of the tool coating or cutting parameters used. TiN tools, which have a lower coating hardness, gave lower hole circularity at the entry and higher cylindricity, while TiN/TiAlN and TiAlN seemed to be more effective in reducing hole particularity when drilling at higher spindle speeds. Finally, optical microscopes revealed that a built-up edge and adhesions were most likely to form on TiN-coated tools due to TiN’s chemical affinity and low oxidation temperature compared to the TiN/TiAlN and TiAlN coatings.

## 1. Introduction

Aluminum and its alloys are used in many industries, including automotive, building, electrical, and aerospace, owing to characteristics such as low metal density (lightweight), durability, electrical conductivity, high strength, corrosion resistance, ductility, and low cost compared with other metals. Aluminum demand is growing worldwide and is expected to grow significantly due to the increased demand as various industries move toward lightweight materials [1,2]. Al6061 is one of the most widely used aluminum alloys in the industry. Its list of applications includes [2]: building material (wide roof structures), welded assemblies, automobiles, aircraft and truck frames, chemical equipment, electronic components, fasteners, yacht building, boats and bicycle frames, and camera lenses. Al6061 consists mainly of aluminum, magnesium, and silicon. The other metallic elements include, in descending quantity order: iron, copper, chromium, zinc, manganese, and titanium [3,4]. Machining of aluminum alloys can be economically, effectively, and efficiently done because their machining properties are superior to those of pure aluminum due to the unique metallurgical structure [2,4,5].

Machining Al6061 and other alloys may pose some challenges, including the wear of the tools. However, it is advisable to use a feed rate up to twice that used for drilling steel for the ease of penetration of most aluminum alloys, given the feed varies with drill diameter. Twist drills are perhaps the most widely used cutting tools for hole-making operations [6]. A good cutting tool should be able to reduce the likelihood of chip adhesion and burr formation to improve the quality of the machined surface. Carbide tools are used for rapid hole drilling due to their prolonged life compared to tools made from high-speed steel and higher hardness [7,8]. The microstructure of the cutting tool was previously reported to influence the machined part’s surface finish and cutting forces when machining Al6061 alloy [9]. Coolants can be an excellent choice to improve part finish when machining light alloys and to prolong tool life [10,11], but this is not always possible, depending on the final part requirements. The choice of feed rates and cutting speeds depends on the workpiece’s mechanical properties, the material of the cutting tool, and its coating. Previous studies [12,13,14,15,16,17,18,19,20,21,22,23] used PCD (polycrystalline diamond), coated and uncoated HSS (high-speed steel), and carbide tools for drilling aluminum alloys. Carbide tools showed superior results in terms of surface finish and prolonged tool life, especially under dry cutting conditions [22]. In addition, coated carbide tools provided lower surface roughness compared to uncoated ones [17]. PCD cutting tools were superior in terms of minimizing adhesion and were most suitable for cutting aluminum alloys under dry machining due to their low coefficient of friction (COF) and low chemical affinity with aluminum [23,24]. On the other hand, a built-up edge (BUE) on the cutting tools is common when machining aluminum alloys [23]. The BUE layer formed on the tool surface comprises aluminum alloy and its precipitates.

Table 1 summarizes the main findings in several studies that investigated the influence of different tool properties on the quality of machining for Al6061 alloy with and without reinforcements. In this table, different analysis methods also were utilized, and their effectiveness to identify the optimum operating conditions was evaluated. The first study in Table 1 [21] can be taken as an example of the effect of different cutting parameters, such as cutting speed, feed rate, point angle, clearance angle, and drill diameter, on machining quality represented by surface roughness, burr thickness, and circularity variance. This study highlighted the effect of cutting speed over other input parameters. The second study in this table [19] showed that Grey relational analysis is an effective method to find the optimum operating conditions. The third study [13] found that feed rate is the most affecting factor in burr formation. Table 1 also shows some studies [13,14,15,19,21,25] that investigated the influence of input parameters, such as spindle speed, feed rate, drill diameter, coating and Al6061 reinforcements, on machining quality and tool defects. For Al6061, Al6061-SiC, and Al6061/20%SiCp, the feed rate was found to be the main parameter that affects hole drill efficiency when the drill tool is HSS or solid carbide [19,25]. For drilling hybrid Al-6061/SiC/B4C/talc using HSS, Kumar et al. [15] stated that cutting speed had a significant impact on the surface-roughness values, and consequently, the surface roughness values were further found to impact negatively on wear of the tool, the drill bit angle, and the cutting speed. The dynamic impact of both feed rate and cutting speed also varies with material reinforcement, material alloys, or components, and tool material and size of the cutting tool [12,21,26]. The most effective parameter may vary according to the reinforcement and the considered machining quality, including the effect on the tool’s surface. It can be concluded from the reviewed studies that the interaction between the workpiece and the tool’s surface is of great importance and improving the tool’s surface may have a considerable effect on the outcome of the machining process. Accordingly, many improvements have been applied to the HSS and solid-carbide drill tools, including various types of coatings. The wide range of coatings and operating conditions that can be used for various workpiece materials encourage research in this field, which contributes to the knowledge in the field of machining. It was also observed that in most machining studies, design of experiments and optimization techniques were usually used to analyze and optimize the measured responses [27]. Optimization is carried out by studying the input parameters of a drilling process such as its tool geometry, coating, cutting parameters, or other related machining parameters that might affect the performance of the analyzed outputs [28].

Surface roughness as a statistical measurement for the characteristics of surface asperities can be of great importance in the manufacturing and machining processes. Higher roughness may reflect asperities of deeper micro-valleys, which in turn results in higher stress concentration and thus increases the possibilities of microcrack initiation [29]. Accordingly, and due to the fracture mechanics theory, surface roughness is related to the fatigue performance and the life of the mechanical component [30]. For the drilling process, one of the quality measurements for the drilled holes is to check the value of surface roughness, which should be within the accepted range [31]. The variation of machining parameters affects the surface roughness of the machined part according to the different vibration, heat, and traction forces generated, which are also affected by the tool’s material, geometry, and surface properties or coating. Accordingly, the operating parameters should be optimized to obtain the lowest roughness. Surface roughness can be represented by many amplitude parameters, such as *R*_a_ and *R*_z_, which are used in the current study. Different factors, such as the cutting-tool material, coating, and cutting parameters can influence the tool life and vibration during drilling, which in turn will affect the form and dimensional tolerances of the hole and cause it to deviate from the perfect circular shape [32].

Achieving the required drilling diameter accurately and reducing the hole circularity reflect the quality of the drilling process. Perpendicularity and cylindricity are important geometrical tolerances that could cause assembly problems, such as fluttering at high pressure in a nozzle check valve, even though other surface tolerances, such as roughness, are within the design limits [33]. The perpendicularity error describes how much the axis of the drilled hole deviates from the normal to the datum surface of the workpiece surface [34]. The perpendicularity error can be measured by the angle between the hole’s axis and the normal to the datum surface. Another widely used perpendicularity measurement is the tolerance between the drilled hole and the maximum pin size to be fitted in the hole normal to the datum surface. The cylindricity error, or tolerance, can be defined as the minimum radial distance separating two coaxial cylinders fitted to the cylindrical surface under examination [33].

It was observed that many of the reviewed studies investigated the effect of cutting-tool coatings on machining Al6061 alloy. Nevertheless, there was no single study that evaluated the effect of the cutting-tool coating when all other cutting parameters were fixed (i.e., all cutting tools had the same diameter, point and helix angles, hardness, tolerance, etc.) on hole quality in Al6061 alloy. Accordingly, this study aims to fill this gap in the field of cutting-tool coatings and assess the influence of drilling parameters (spindle speed (*n*), feed rate (*f*)) and the type of tool coating (TiN/TiAlN, TiAlN, and TiN) on hole-quality metrics (surface roughness, hole-size circularity, cylindricity, and perpendicularity) during drilling of Al6061 alloy. Moreover, the current study evaluates the different forms of damage that may form on the surface borehole in Al6061 alloy when drilling using those three types of tool coatings, and the mechanisms responsible for their formation. The results of the drilling experiments were further evaluated using the statistical method ANOVA (analysis of variance) to assess the percentage contribution of each input parameter and their linear effects on the analyzed outputs.

**Table 1:** Summary of reviews of research publications on the impact of machining parameters on machining quality of aluminum alloys.

Material	Tool Details	Input Parameters	Analyzed Outputs	Refs
Al6061 drilling	Uncoated HSS Helix angle: 45° Point angle: 100°, 110°, 118° Drill diameter: 8, 10, and 12 mm	Feed rate: 0.3, 0.5, 0.6 (mm/rev) Spindle speed: 600, 800, 1000 (rpm)	SR, BH, BT, CIRC	[19,21]
AI6061 drilling	TiN-HSS Point angle: 118° Drill diameter: 8 mm	Feed rate: 0.04, 0.08 (mm/rev) Spindle speed: 1000, 1500, 2000 (rpm)	CF, TW, BH, BT, CIRC, CHF	[13]
Al6061/20%SiCp composite drilling	Tipped carbide Point angle: 90°, 118°, 135° Drill diameter: 10 mm	Feed rate: 0.12, 0.16, 0.20 (mm/rev) Spindle speed: 900, 1120, 1330 (rpm)	CF, SR	[20]
Al6061/B4C composite milling	Uncoated carbide inserts Cutting diameter: 40 mm	Feed rate: 0.08, 0.1, 12, 0.16 (mm/tooth) Milling speed: 220, 285, 370, 480 (mm/min)	SR, PO	[16]
Al6061-T6 drilling	Uncoated HSS Drill diameter: 11.7 mm	Feed rate: 0.2, 0.3, 0.4 (mm/rev) Cutting speed: 60, 75, 100 (rpm)	HS, CIRC, SR	[26]
Al6061/SiC/B4C/t lc composite drilling	Uncoated HSS Drill diameter: 6, 7, 8 mm	Feed rate: 15, 25, 35 (mm/min) Cutting speed: 750, 1000, 1250 (rpm)	CF, SR, CIRC	[15]
Al2124/20 % B4C end milling	Uncoated carbide	Feed rate: 0.1, 0.2, 0.3 (mm/rev) Cutting speed: 50, 100, 150 (rpm)	SR	[14]
Al6061 and Al6061-SiC drilling	Uncoated carbide Point angle: 96°, 118°, 140° Drill diameter: 10 mm	Feed rate: 0.1, 0.15, 0.2 (mm/rev) Cutting speed: 40, 60, 80 (rpm)	BH, BT	[25]
Al6061-T6 turning	-	Feed rate: 0.2, 0.1, 0.05, 0.01 (mm/rev) Spindle speed: 1000 (rpm)	SR (rendering)	[35]

Note: SR: surface roughness; BH: burr height; BT: burr thickness; CIRC: circularity; CF: cutting forces; TW: tool wear; HS: hole size; CHF: chip formation; PO: power.

## 2. Materials and Methods

### 2.1. Workpiece Material

Al6061-T651 was used in the current study. The material is an aluminum alloy hardened by precipitation, containing Mg (magnesium) and Si (silicon) as its main alloying components. Table 2 shows detailed information on the mechanical, electrical, and thermal properties. The size of the Al6061-T651 workpiece used in this work was 210 × 150 × 10 mm^3^. Three drilling tools with three different coatings (TiN/TiAlN, TiAlN, TiN) with a 10 mm diameter were used. For each type of coating, two parameters (spindle speed and feed rate) were tested with three levels for each parameter, thus requiring the drilling of 27 holes. However, to reduce any scattering in the results, each drilling was repeated three times, and thus the total number of holes drilled in this study was 81. Accordingly, 9 × 9 holes were drilled in the workpiece with a center-to-center distance of 15 mm between adjacent holes in each row, and a 15 mm distance between the adjacent rows. These distances were chosen to ease the drilling process and minimize the effect of the drilling on adjacent holes in the workpiece. The hardness of the workpiece was measured using a Vickers hardness tester (Köseler, Istanbul, Turkey) using a 30 Kgf pyramid indenter, and was found to range between 111.5 and 112.5 HV.

### 2.2. Cutting Tools

Carbide-coated twist drills 10 mm in diameter with a point angle of 140° and a helix angle of 30° were used in the current study. For most drills, the standard helix angle is usually 30° [6], although the majority of drills have a 118° drill point angle, and the advised point angles for drilling operations on Al6061-T651 alloy materials are within 130°–140° [12,13,14,15,19,25,36,38,39,40]. Additionally, large helix angles, typically greater than 24°, allow rapid evacuation of chips, whereas large point angles boost chip evacuation and minimize burrs. The silicon content in the aluminum alloy governs the point angle of the drill. For example, a point angle of 130°–140° is recommended for aluminum alloys with low or no silicon content [12,13,14,15,19,25,36,38,39,40] It has also been reported that point and helix angles affect the surface roughness, such that increasing these parameters can minimize burr formation and improve surface quality [13]. In addition, the 10 mm diameter drill bit was selected, as it is a regular size in Al6061 applications for creating holes, and this hole size allows the insertion of the stylus probe for the surface-roughness tester inside the hole.

**Figure 1:**
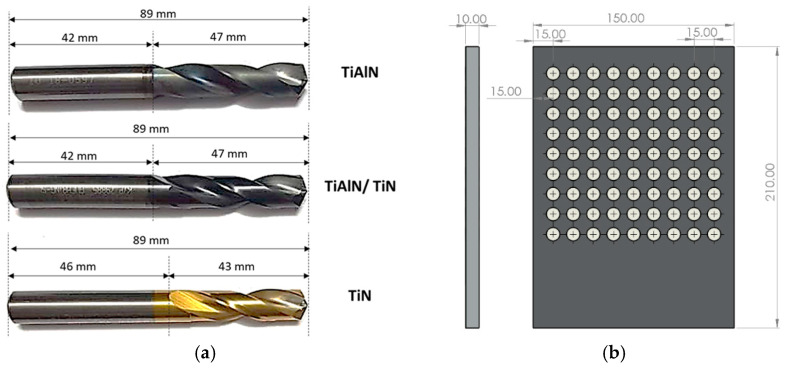
Details of (**a**) side view of the three types of cutting tools used in the study, and (**b**) plate dimensions and hole-array setup.

Figure 1 shows geometrical details of the drilling bits and the workpiece. Table 3 shows the three types of tool coatings investigated in this study. Firex coating is the multilayer coating of titanium aluminum nitride (TiN/TiAlN) applied to carbide drills. It provides maximum wear resistance and high thermal stability during drilling operations, and it is more suitable for dry machining. According to previous studies [12,41], firex coating performs better than TiN coating; combines the benefits of TiN, TiAlN, and TiCN; and resists fire and heat. Titanium nitride (TiN) coating is commonly used for general cutting of metals and plastics [12]. TiN coating has high ductility and can protect the cutting tool from abrasive and adhesive wear [12,42]. The good thermal stability and low COF of TiN coating lower burrs and improves the heat removal from the cutting zone. Titanium aluminum nitride (TiAlN) coating also has good ductility and is more suited for dry-machining applications; it has an enhanced oxidation resistance and higher hardness than TiN coating [12,41].

### 2.3. Experimental Procedure

Drilling tests were carried out on a Rapimill 700-CNC milling machine (Knuth, Neumünster, Germany) that provides spindle speeds of up to 7000 rpm. The Al6061 sample was mounted by machine work-holding fixtures and held in place. The drilling parameters used in the current study are shown in Table 4. To validate the study’s repeatability, each combination of experimental parameters was repeated three times, and their mean values were reported thereafter. To detect the effect of input parameters (i.e., spindle speed, feed rate, and tool coating) on measured outputs, the analysis used a full factorial design with three variables and three levels, as shown in Table 4. The surface-roughness metrics and hole form and dimensional tolerances were analyzed using ANOVA in MINITAB 18 software (18.1, State College, Centre County, PA, USA) to check their contributions and the interaction of the input parameters on the analyzed outputs.

For each type of tool coating, a fresh drill was used to drill a set of nine holes combining three feed rates and three spindle speeds to minimize any effects arising from tool wear [12]. The drilling tests were conducted under dry conditions. The cutting parameters were chosen based on previous literature on the machining of metal alloys and tool manufacturers’ recommendations. As evident from the past literature, the typical feed rate for drilling aluminum alloys ranged between 0.05 and 0.3 mm/rev, with spindle speeds of 1000 to 10,000 rpm [6,18,29] depending on the size of the cutting tool, grade of the aluminum alloy, and analyzed outputs.

### 2.4. Measurement of Surface Roughness

The arithmetic height average *R*_a_ is one of the most widely adopted measurements for surface roughness. *R*_a_ is the average of the absolute height deviation from the mean line over sampling length, as shown in Equation (1) [43]. The 10-point height roughness parameter *R*_z_ was also used in the current study, as it is more sensitive than *R*_a_ to irregular heights or depths of the peaks and valleys, respectively. According to the international standard ISO, *R*_z_ is the difference between the average of the five maximum heights and the average of the five lowest valleys over the measured length, as shown in Equation (2) [43].

(1) $$R_{a}=\frac{1}{l}\int_{0}^{l}\left|y\left(x\right)\right|dx\;\;\equiv \;\frac{1}{n}\overset{n}{\underset{i=1}{\sum }}\left|y_{i}\right|$$

(2) $$R_{z\left(\mathrm{ISO}\right)}=\;\frac{1}{n}\left(\overset{n}{\underset{i=1}{\sum }}p_{i}-\overset{n}{\underset{i=1}{\sum }}v_{i}\right)$$

where *y*(*x*) is the profile function, *p_i_* is the peak points, *v_i_* is the valley points, and *n* is the number of samples.

The roughness parameters *R*_a_ and *R*_z_ were measured using a Mitutoyo SJ-210 surface-roughness tester (Mitutoyo, Kawasaki, Japan). The device has a measuring range of 17.5 mm and a detector range of 360 µm (−200 µm to +160 µm). It also has a measuring force of 4 mN and a 5 µm stylus diamond tip. The driving speed of the stylus was kept constant at 0.5 mm/s during the measurement. The Al6061 workpiece was placed vertically, with the axes of the holes in the horizontal direction [12,38]. The roughness tester was placed in front of each hole to allow the stylus arm, which has the probe tip, to be inserted inside the holes. The overall travel length was set to approximately 10 mm. For each hole, four measurements were taken its periphery at 0°, 90°, 180°, and 270°, and their average was reported for each hole-roughness value (*R*_a_ and *R*_z_).

In addition, the holes were cut in half and the wall of the hole was examined using a Zygo Zegage contactless surface profilometer (Zygo ZEGAGE, Zygo Corporation, Middlefield, CT, USA). In addition, ZeMaps and MetroPro surface-analysis software (version, Zygo corporation, CT, USA) were used to monitor the surface topography and further analyze the surface roughness of the holes. From this, S_a_, which is the extension of *R*_a_ (arithmetical mean height of a line) to a surface, and ISO SR_Z_ which is the extension of R_z_ were measured to evaluate the surface roughness metrics for certain areas of interest on the borehole surface (below hole entry, at the middle portion of the hole, and near the hole exit). S_a_ and ISO SR_Z_ were measured by scanning an area from the surface using an optical profiler. As stated by the manufacturer, the texture results obtained from the instrument comply with the ISO 25178 standard. Therefore, S_a_ and ISO SR_Z_, which are reported in the aerial graphs, reflects the surface texture of all points in the scanned area, rather than a profile.

### 2.5. Measurement of Hole Form and Dimensional Tolerances

**Figure 2:**
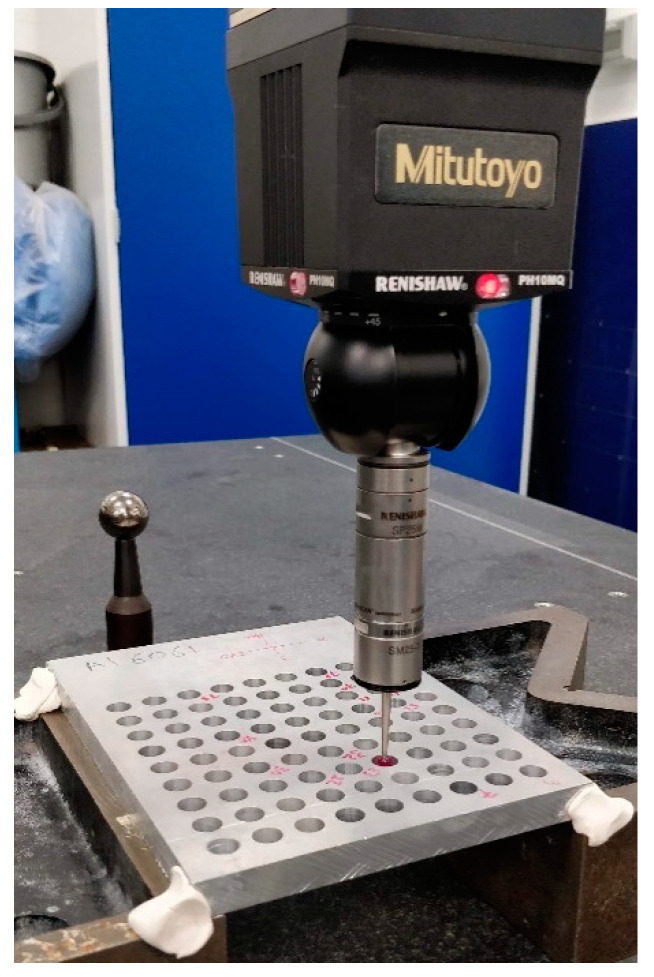
Al6061 workpiece setup inside the CMM machine.

A coordinate-measuring machine (CMM) traces the profile of the hole using a stylus or probe to map the coordinates of a specific number of points on the perimeter of the hole. In the current study, the hole form and dimensional tolerances were measured using a Mitutoyo CMM machine equipped with a RENISHAW PH10MQ head (Renishaw, Gloucestershire, UK), as shown in Figure 2. The workpiece with the 81 holes was placed horizontally on the machine table and the measurements were taken at two levels, which were called top and bottom; they were at 1 mm below and above the upper and lower surfaces of each hole, respectively.

### 2.6. Scanning Electron Microscopy

Scanning electron microscopy was carried out to analyse the borehole surface quality throughout its thickness. A Hitachi SU5000 field emission scanning electron microscope (SEM, Hitachi, Chiyoda, Japan) was used to scan a portion of the hole after it was cross-sectioned. The SEM scans were taken at the entry and the exit of each hole, as well as the overall borehole surface.

## 3. Results and Discussion

Table 5 shows the percentage contribution of the cutting parameters (i.e., spindle speed, feed rate) and tool coating on the analyzed outputs. The data was extracted from the ANOVA analysis carried out in Minitab software. This table will be used in the following sections to support the discussion of the observed results. The values in red font color are those with significance in the statistical ANOVA model, i.e., *p*-value < 0.05.

### 3.1. Analysis of Surface-Roughness Metrics

**Figure 3:**
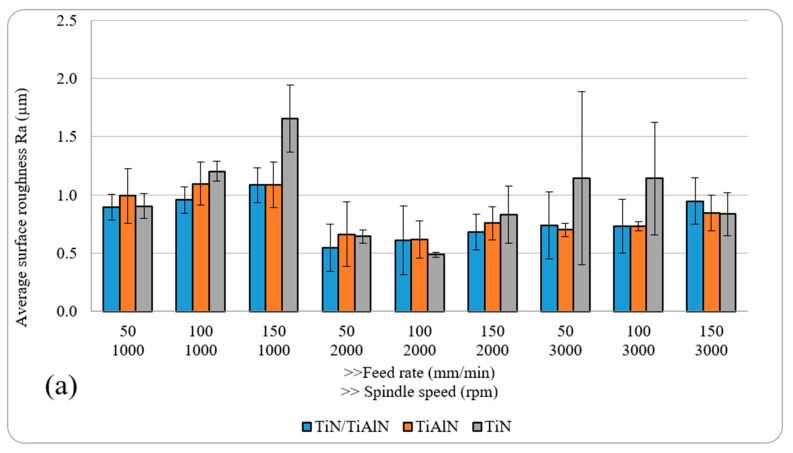

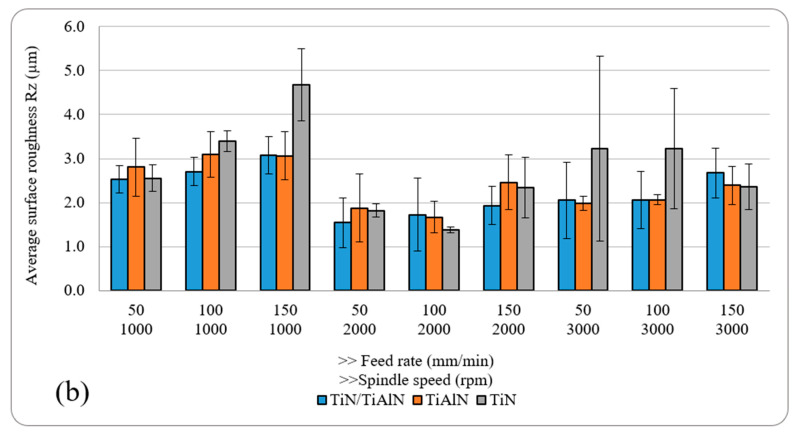
Average hole surface roughness: (**a**) *R*_a_; (**b**) *R*_z_.

Figure 3 shows the results of the surface roughness (*R*_a_ and *R*_z_) for holes drilled with different cutting parameters and tool coatings. Generally, *R*_a_ ranged between 0.49 µm and 1 µm, while *R*_z_ ranged between 1.38 µm and 4.68 µm. The results of surface roughness *R*_a_ reported here were around 30–40% lower than those reported by Aaamir et al. [44], who drilled holes in Al6061 alloy using a similar range of cutting parameters and 6 mm uncoated carbide twist drills. This could indicate that increasing the drill diameter does not lead to an increase in the surface roughness. Moreover, this also confirms that coated tools in general have a positive effect in reducing the surface roughness. The results also showed that the surface-roughness metrics *R*_a_ and *R*_z_ were influenced by both cutting parameters. For example, *R*_a_ and *R*_z_ increased with the rise of the feed rate for the TiN/TiAlN and TiAlN coatings, which was mainly due to the increase in uncut chip thickness. Similar trends were observed in holes drilled using TiN-coated tools when drilling at *n* = 1000 rpm. Increasing the spindle speed tended to decrease due to the reduction in uncut chip thickness and increasing the effect of ploughing, rather than cutting with chip formation with increasing the spindle speed. Increasing the spindle speed further caused an increase in both roughness metrics, which was attributed to the increased deformations and cutting temperatures at higher spindle speeds. The highest and lowest *R*_a_ and *R*_z_ were found in holes drilled using TiN-coated tools (highest *R*_a_ and *R*_z_ at *n* = 1000 rpm and *n* = 150 mm/min, lowest *R*_a_ and *R*_z_ at *n* = 2000 rpm and *f* = 100 mm/min). Similarly, the lowest *R*_a_ and *R*_z_ for the other two coatings was also observed to occur under *n* = 2000 rpm, which could indicate that this spindle speed is most suitable for drilling Al6061 alloy with minimal surface roughness.

Generally, it is not possible to make a firm conclusion on which coating provided the lowest surface roughness. This is mainly because of the relatively similar surface-roughness results obtained from the three coatings. Indeed, previous studies that compared the performance of TiN- and TiAlN-coated tools for machining aluminum alloys found that they produced similar roughness results [45]. However, it was observed that holes drilled using TiN-coated tools showed a somewhat greater deviation and the highest recorded roughness values, despite TiN coating having a slightly lower COF than the other two coatings. This could be related to the lower thermal stability and heat resistance under dry-machining conditions. According to Table 3, TiN coating has a much lower oxidation resistance and hardness compared to TiAlN and TiN/TiAlN coatings, which could adversely impact surface-roughness metrics. It was also found that the surface roughness in some of the holes drilled using TiN-coated tools was higher than in those drilled using the other two coatings at the same cutting parameters. This could be attributed to the coating’s chemical affinity to interact with the alloy, causing chips to diffuse and adhere to the cutting-tool surface, therefore increasing the built-up edge and further increasing the surface roughness of the machined holes. Some of the cut aluminum chips would not escape the cutting zone and ended up binding with the coating on the cutting-tool surface, aluminizing the drill-bit surface and raising the contact friction between the drill and the material, which further increased the hole surface roughness [12]. It should be also noted that the repeatability of surface roughness, in general, was low; this, in turn, affected the ANOVA results, which showed that only the spindle speed had a significant effect on the surface-roughness metrics (about 26–27.5%), while the feed rate and the cutting-tool coating or their linear interactions did not have any effect.

**Figure 4:**
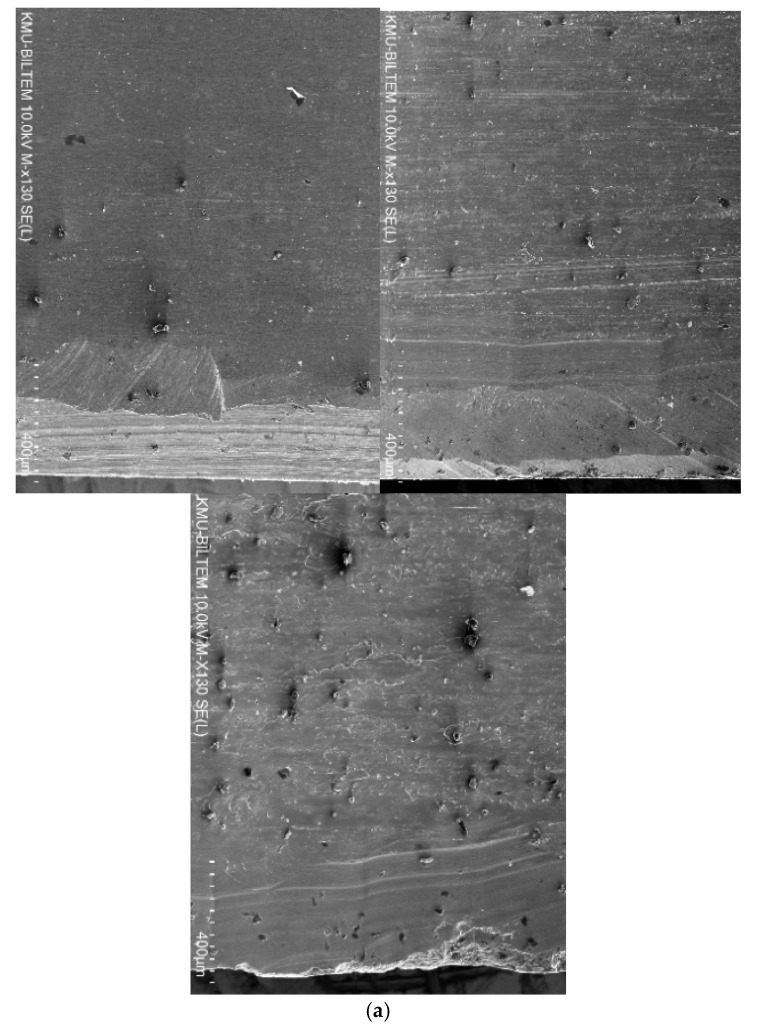

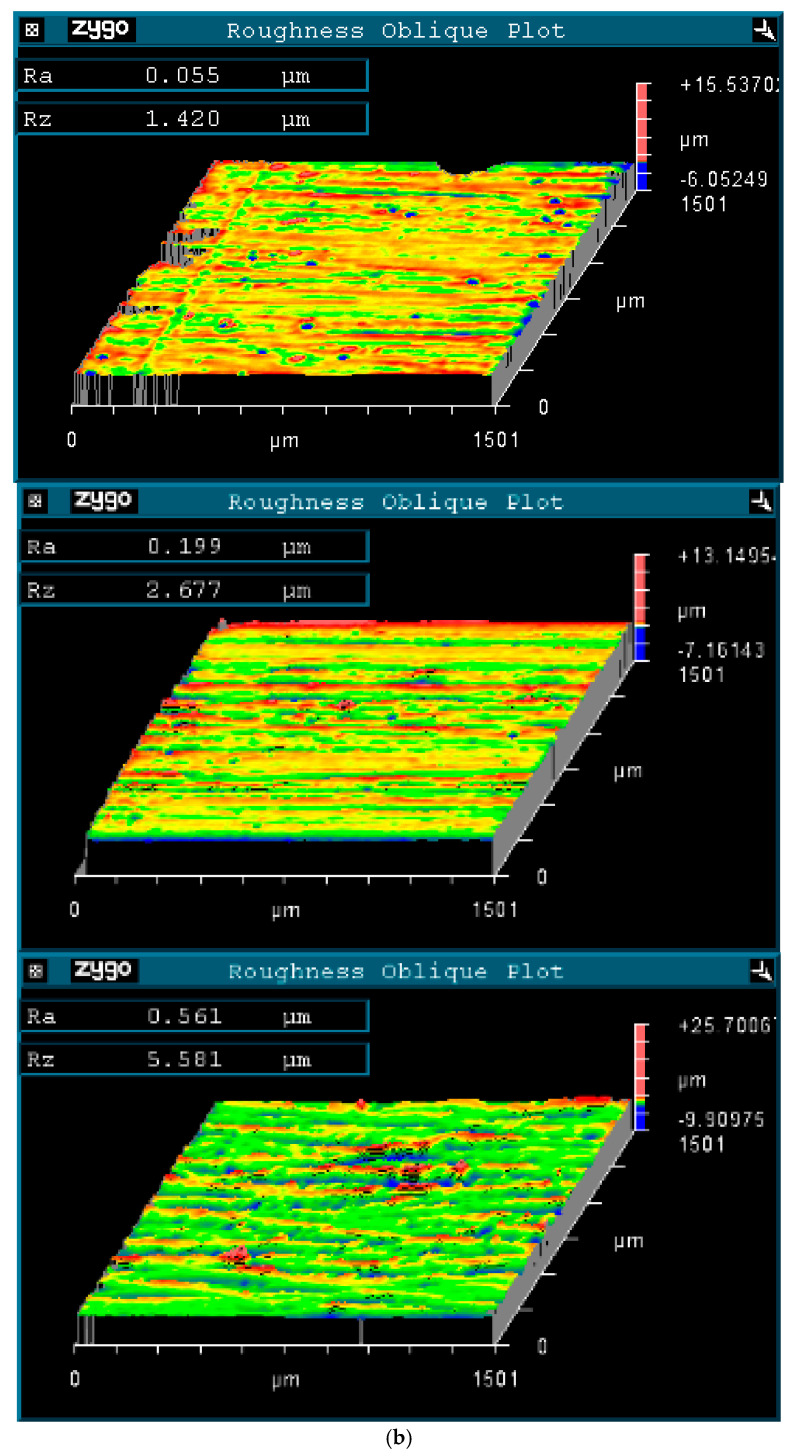
Hole condition showing (**a**) quality of holes at the exit, and (**b**) 3D surface-roughness topography at the exit showing average *R*_a_ and *R*_z_ values (from left to right: using TiN/TiAlN, TiAlN, and TiN coating).

It is important to notice that with a 2D roughness measurement using a mechanical stylus, only specific lines along the hole path from entry to exit are checked for roughness quality, while a 3D surface analysis provides a broader area evaluation for surface-roughness metrics. However, a limitation with using 3D surface-roughness techniques is the small area that can be evaluated for roughness, which requires taking many measurements along the hole depth and is time-consuming. Therefore, further surface analysis was carried out using 3D optical microscopy. The roughness measurements were taken at three distinct locations, as stated earlier: below the hole entry, at the middle portion of the hole, and above the exit side. At the hole entry, it was observed that holes drilled using TiN-coated tools at *n* = 1000 rpm produced the worst surface-roughness finish for both *R*_a_ and *R*_z_, which were also found to increase with the increase of the feed rate. At the middle portion of the hole and its exit, the surface roughness in holes drilled at *n* = 1000 rpm using TiN-coated tools was higher than those drilled using the other two types of tool coatings. Holes drilled using TiN/TiAlN-coated tools showed a somewhat better surface finish than those found in holes drilled using the other two types of tool coatings, as shown in Figure 4. This was mainly attributed to less damage and debris observed on the borehole surface.

**Figure 5:**
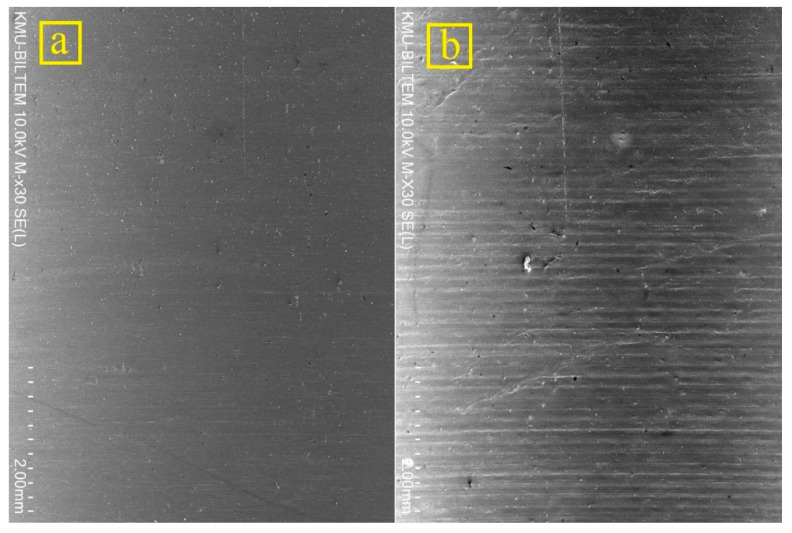
Comparison of hole surface quality in a hole drilled using TiN-coated tools at (**a**) *n* = 2000 rpm and *f* = 150 mm/min, and (**b**) *n* = 3000 rpm and *f* = 50 mm/min.

However, since only one set of holes was evaluated for each type of tool coating, these can be considered as observations for the hole quality in that set, and cannot be used to make a conclusion on which coating gave the best surface finish. It was also observed that all holes drilled at *n* = 3000 rpm and *f* = 50 and 100 mm/min tended to produce the worst surface finish, regardless of the type of tool coating used. This would indicate that these sets of cutting parameters are not suitable for drilling Al6061 alloy, and should be avoided if surface roughness is to be minimized. In addition, under all tested parameters, it appears that drilling at *n* = 2000 rpm and *f* = 100 and 150 mm/min somewhat gave the lowest surface-roughness metrics *R*_a_ and *R*_z_, regardless of the type of tool coating used, as can be seen in Figure 5.

**Figure 6:**
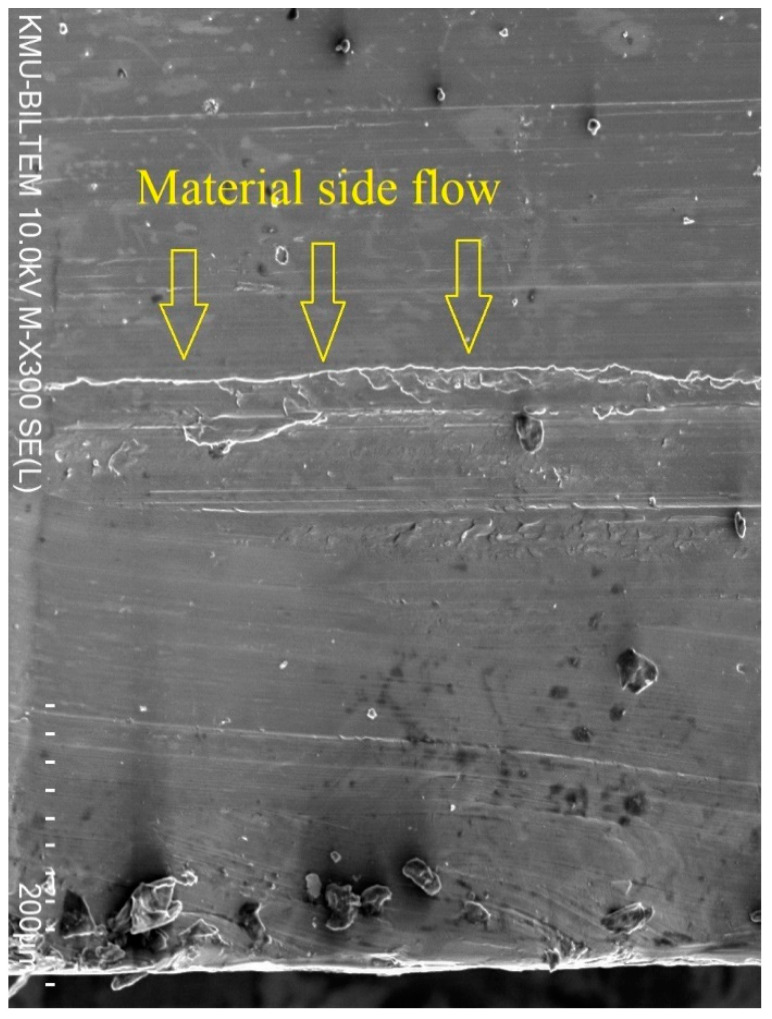
SEM image of hole at exit side drilled using TiN-coated tools, showing the material side flow phenomena.

This could be attributed to the phenomenon of material side flow, which can significantly deteriorate the machined surface quality and can be seen in Figure 5b. Material side flow, which is the displacement of workpiece material in a direction opposite to the feed direction, occurs due to the plastification of the workpiece material during the machining process as a result of high cutting temperatures and pressure. The material side flow is highly influenced by the cutting-tool geometry and characteristics, and increases when the tools have lower thermal stability and hardness, such as the case with TiN-coated tools when compared to TiN/TiAlN- and TiAlN-coated tools, due to increased changes in tool geometry during the drilling process [46]. Kishawy et al. [47] previously reported that the material side flow at the feed marks is mainly attributed to the trailing edge wear where material plastic flow fills the groove, and the excess material is pressed to the side of the tool. A close examination of the borehole surfaces drilled using different cutting-tool coatings shows that extensive material plastic flow was more likely to occur when drilling using TiN-coated tools, as can be seen in Figure 6.

To summarize, the analysis of the influence of cutting parameters and tool coatings on *R*_a_ and *R*_z_ indicates that intermediate spindle speeds and low feed rates give a lower surface roughness regardless of the cutting tool coating used. This conclusion also agrees with a previous study on drilling hybrid aluminum composite material [12]. In addition, the dry drilling of Al6061 laminates with the tool coatings investigated in this study led to a range of surface roughness for *R*_a_ between 0.49 µm and 1 µm. Looking at past studies in the open literature and technical documents, it is clear that there is no fixed value of surface roughness that is recommended for the acceptable surface finish of a machined hole. As a rule of thumb, a lower surface finish is always desirable to reduce the fatigue and corrosion effects on metals in general [48]. Some technical reports recommend that hole surface roughness *R*_a_ be less than 1.6 μm in aluminum parts if used in critical applications, such as in the aerospace industry [49,50]. This means that the roughness results obtained in this study were within the recommended values, and similar or better than those reported in the previous literature on machining Al6061 alloy under dry conditions [15,16,20,21,40]. Another interesting conclusion is that previous studies showed that TiAlN coating tends to give better or equivalent surface roughness when machining aluminum alloys under dry conditions compared to other coatings, including TiN-coated tools [38,51,52,53], which agrees with what was found in the current study.

### 3.2. Effects of Cutting Parameters and Tool Coatings on Hole Size

**Figure 7:**
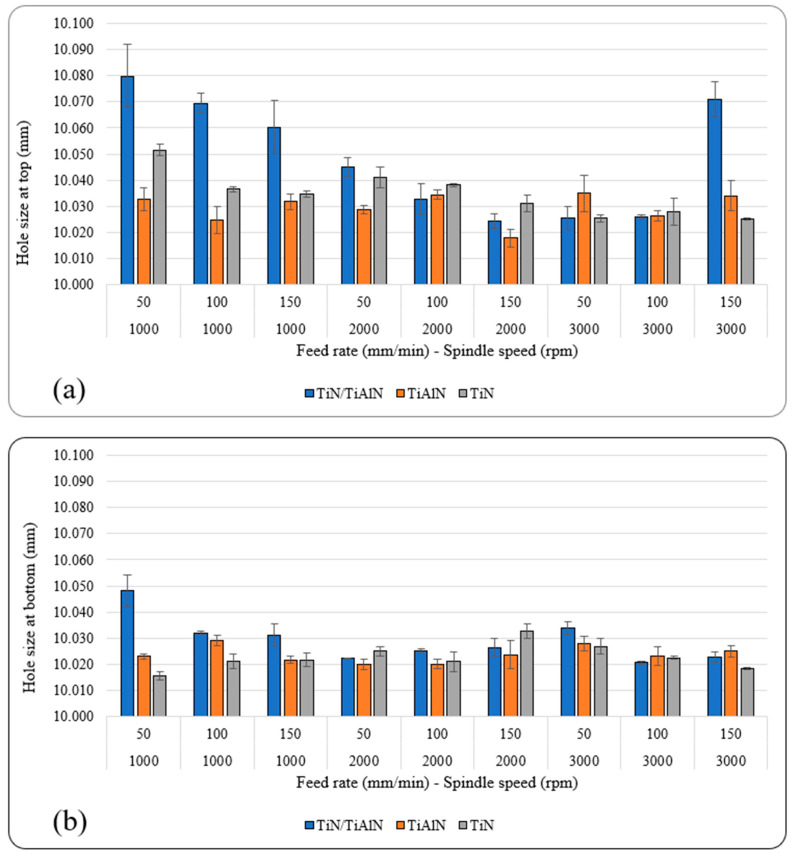
Average hole size: (**a**) at the top of the hole; (**b**) at the bottom of the hole.

Figure 7a,b show the average hole size at the top and bottom of each hole under different spindle speeds, feed rates, and tool coatings. The results indicated that all holes were oversized at the two measured locations throughout the hole. This means that drilling Al6061 alloy using carbide drills would always produce oversized holes regardless of the tool coating used. This was also reported in previous studies [40,49,54], which found that oversized holes were always produced when drilling aluminum alloys.

The hole size at the bottom was always smaller than that at the top under all cutting parameters and tool coatings used. This is possibly due to the drill “wander” on contact with the workpiece, where it is point-supported, then at further depth, where it is point- and side-supported [39,54]. Vibratory displacement occurred during the initial contact between the chisel edge and the workpiece (i.e., hole entry), which triggered dynamic instability, causing higher hole-size deviations at the top than at the bottom [39,45]. Hole size in this study ranged between 15.47 µm and 79.9 µm, as shown in Table 6.

**Figure 8:**
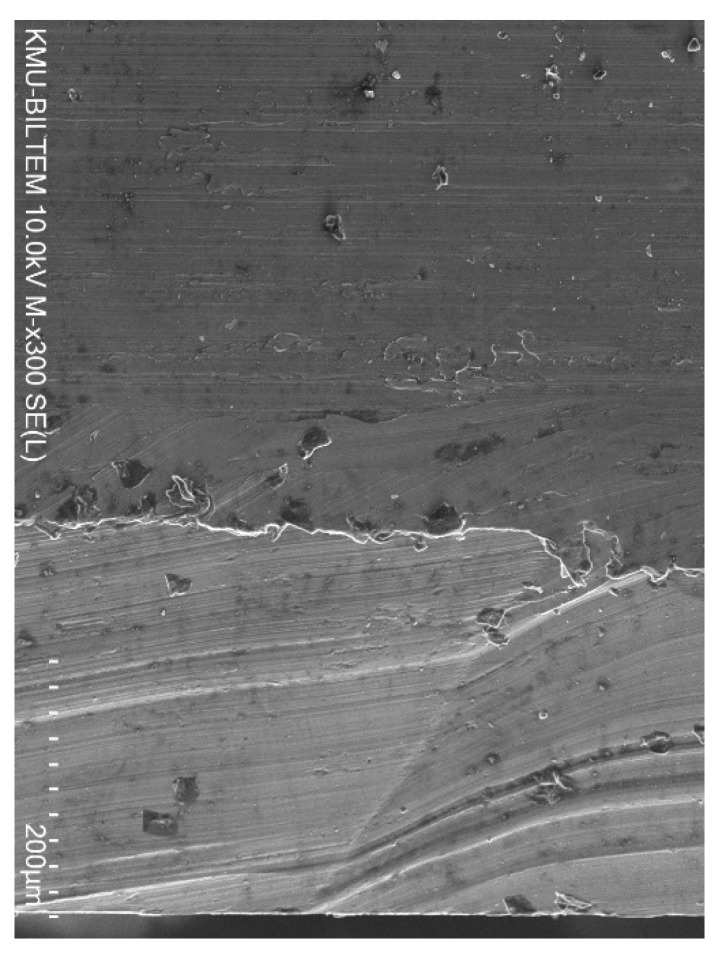
SEM image of hole drilled using TiN/TiAlN-coated tool at the exit, showing the formation of the white layer.

Generally, TiAlN-coated tools produced holes with the least deviation at the top and bottom. This could be due to the higher nano-hardness of the cutting tools, which maintains higher rigidity during the drilling process relative to the other two coatings. The largest deviation in hole size occurred at *n* = 1000 rpm, for holes drilled using TiN/TiAlN-coated tools at the top and bottom locations. Also, holes drilled using TiAlN-coated tools gave the lowest deviation at the top of the hole, and holes drilled using TiN-coated tools showed the lowest deviation at the bottom of the hole. This could be attributed to the formation of the white layer at the exit side of the hole drilled using TiN/TiAlN-coated tools (Figure 8), which might have had some influence on increasing the hole geometrical metrics. During the drilling process, the cut chip is removed from the workpiece by the action of the cutting tool, while some of the uncut chip reaches a high temperature and is melted then resolidifies on the borehole surface, forming a new layer. This is caused by the severe plastic deformation and high machining temperatures that occur during the drilling process. The resolidified material is usually observed to exist near the hole exit surface; it reattaches to the surface after the tool exits the workpiece from the other side, forming a layer known as the white layer [55]. The white layer may contain elements from the tool-coating material; this will be the scope of a future study. It was observed that a significant presence of white layers was found to form at the exit of the holes drilled using TiN/TiAlN-coated tools, and its thickness was 200 µm on average and reached up to 400 µm. The white layer was found to increase with the increase of the feed rate, and did not seem to be affected by the spindle speed. The white layers also were found to form on the holes drilled using TiAlN-coated tools when drilling at a high feed rate of 150 mm/min. This would imply that the presence of aluminum in the TiN/TiAlN- and TiAlN-coated tools was responsible for increasing the chemical interaction between the cutting tool and the workpiece, leading to the formation of such white layers.

At *n* = 2000 rpm, the difference in the performance of the three coatings became less noticeable, and varied according to the feed rate; however, the TiAlN showed some marginal lead compared to the other two coatings at the top and bottom of the hole. At *n* = 3000 rpm, the three coatings showed a similar trend, with slightly better performance for holes drilled using TiN coating at the highest spindle speed and feed rate at both locations. It was also found that for holes drilled using TiN/TiAlN- and TiAlN-coated tools, the hole size at the top decreased with increasing feed rate when drilling at *n* = 1000 and 2000 rpm. No other possible trends were possible to deduct from Figure 7a,b. However, ANOVA analysis revealed that the cutting parameters and tool coatings, and their linear interactions, all had a significant influence on hole size at the top and bottom. For example, it was found that the tool coating showed the highest contribution to the hole size at the top and bottom (23.9% and 16.8%, respectively). The spindle speed also had a significant influence on hole size at the top (16.89%), and a minor effect at the bottom (3.75%). Although the feed rate influenced the hole size, it nevertheless was minimal, and ranged between 1.95% at the top and 3.39% at the bottom. The linear interactions between the cutting parameters and tool coatings had a significant effect on hole size at the top and bottom. For example, the interaction between the spindle speed and the tool coating had a 19.24% contribution at the top and 26.99% at the bottom. This indicates that the hole size is a function of spindle speed and tool coating; therefore, to optimize the hole size, it is important to consider proper spindle speeds that would not increase the temperatures at the cutting zone. Therefore, it can be said that the performance of each coating was affected by its mechanical and thermal properties, such as hardness and oxidation resistance, where the advantage was for TiN/TiAlN and TiAlN coatings; and thermal diffusivity, where TiN has the highest, followed by TiAlN and TiN/TiAlN [56]. Overall, it can be said that holes drilled using TiAlN-coated tools produced holes with the least deviation from the nominal diameter. The effect of the feed rate cannot be described by a single trend for all spindle speeds and coatings. However, one of the observed trends was the reduction of hole-size error with increasing feed rate at specific speeds, which can be seen for TiN/TiAlN coating at 1000 rpm (top), 2000 rpm (top), and 1000 rpm (bottom); and TiN coating at *n* = 1000 rpm (top), 2000 rpm (top), and 3000 rpm (bottom). On the other hand, no specific trend could be identified for TiAlN. Similar to the feed rate, no single trend could describe the effect of the spindle speed. However, reducing the hole-size error with increasing the spindle speed was observed for TiN/TiAlN coating at 50 mm/min (top), 100 mm/min (top), 100 mm/min (bottom), and 150 mm/min (bottom); and TiN coating at 50 mm/min (top) and 150 mm/min (top). The opposite trend was observed for TiAlN at 150 mm/min (bottom); and TiN at 50 mm/min (bottom). Figure 7a also shows a reduction in the hole size at the top for TiN coating when increasing the feed rate and spindle speed, but keeping the ratio of the constant as 50/1000, 100/2000, and 150/3000. Also, Figure 7b shows a similar trend for TiN/TiAlN at the bottom of the hole.

To conclude the hole-size analysis, industries such as aerospace have strict demands when drilling metallic structures, as they require an H7 hole tolerance fit (±12 micron deviation from the hole nominal diameter) based on the ISO 286 standard [57]. However, such stringent tolerances are difficult to achieve, and therefore more hole tolerances are relaxed, requiring between H8 (±18 microns) and H9 (±30 microns). Moreover, cutting-tool manufacturers suggest that an acceptable hole-size tolerance in metals such as aluminum alloys can be anything between ±20 and ±40 microns [50,58]. Of course, for automotive applications, the tolerances are further relaxed due to a less-critical impact on the structural integrity of the vehicle. This means that most of the hole-size data found in this study are within the recommended range of hole tolerance required in aerospace and automotive applications, except for holes drilled at low spindle speeds of *n* = 1000 rpm using TiN-coated tools.

### 3.3. Effects of Cutting Parameters and Tool Coatings on Circularity Error

**Figure 9:**
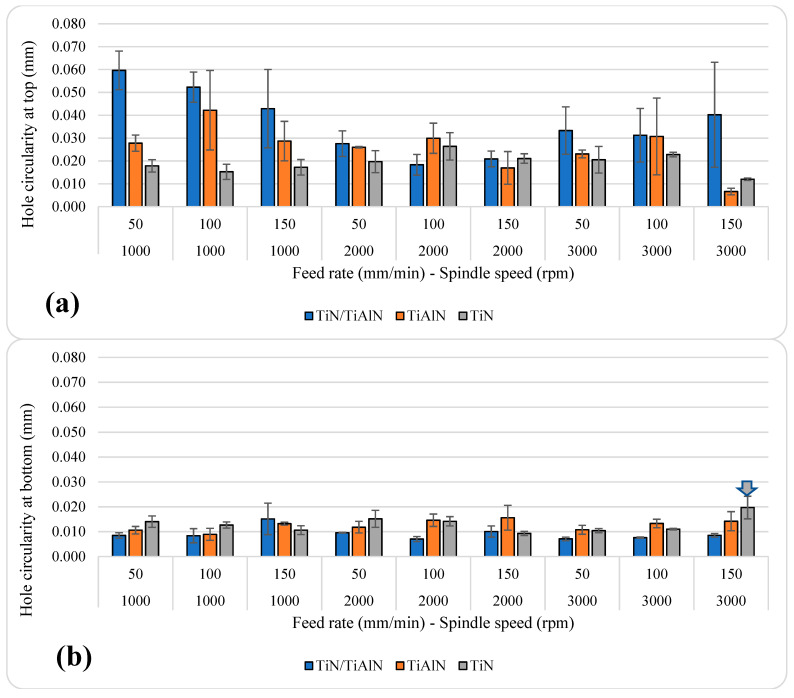
Average hole circularity errors: (**a**) at the top of the hole; (**b**) at the bottom of the hole.

Figure 9 shows the average hole circularity at different feed rates and spindle speeds using the three tool coatings. The results showed a similar trend to that observed for hole size. The circularity error at the bottom of the hole was always less than that at the top of the hole, which may be related to the stability of the tool at different depths. This observation is in agreement with previous work, which reported that when drilling metals, the hole-circularity error at the exit side was lower than at the hole entry due to the dynamic instability of the drill during the first contact between the chisel edge and the workpiece [45,57]. This could be attributed to the weakening of the workpiece structure below the tip of the cutting tool due to the reduction in uncut material thickness from the hole as the drill progressed throughout the hole toward the exit [57]. The reduction in hole circularity with depth in metallic alloys was due to the increased support to the cutting tool by the hole walls, which provides a form of self-pointing guidance action to the cutting tool [26,57,59,60]. Indeed, previous studies indicated that there is a presence of highly nonlinear variations in hole circularity throughout the depth of the hole when drilling aluminum alloys due to other factors such as the fixture setup of the workpiece, the dynamic interaction between the drill and the workpiece, and resulting vibrations in the cutting tool, as well as cutting-tool deflections and damping characteristics [39,54,57]. Such factors were not considered in this work, but will be the scope of a new study in the future.

Overall, the average hole-circularity error ranged between 12 and 59.63 µm at the top; while at the bottom, it was between 7.03 and 19.7 µm, as shown in Table 7. Similar to the hole-size error, the holes drilled with TiN/TiAlN-coated tools showed higher circularity error at the top of the hole for most speeds and feed rates; however, this was not the case on the bottom of the hole, since this coating showed the lowest circularity error among the other coatings. This could be due to the higher oxidation resistance of TiN/TiAlN-coated tools, which becomes critical with hole depth where temperatures are expected to rise.

It also can be said that TiN coating showed somewhat lower hole-circularity errors at the top compared to the other coatings under most cutting parameters. This could be due to the lower COF and lower hardness, which does not cause severe deformations when the hole is in initial contact with the workpiece top surface. In terms of which tool coating gave the lowest hole circularity, in general, a higher feed rate resulted in a higher circularity error on the bottom of the hole. On the top level, no specific trend could be recognized at all spindle speeds. For TiN/TiAlN, the relation between circularity error and feed rate was inverse at 1000 rpm, positive at 3000 rpm, and parabolic at 2000 rpm. The lowest circularity error was observed at the highest spindle speed and feed rate in holes drilled using TiAlN- and TiN-coated tools. To a certain extent, at the top level, the effect of spindle speed can be recognized by a parabolic behavior for TiN/TiAlN. For TiAlN, on the top level, circularity error related almost inversely to the spindle speed. For TiN, the relation was almost positive between spindle speed and circularity error on the top level, while no clear relation could be observed on the bottom level.

Holes drilled using TiN/TiAlN-coated tools showed the highest hole circularity among the three coatings (at *n* = 1000 rpm and *f* = 50 mm/min). Holes drilled using TiAlN-coated tools showed the lowest hole circularity at the top among the three coatings (at *n* = 3000 rpm and *f* = 150 mm/min). Similarly, holes drilled using TiN-coated tools gave the highest hole circularity at the bottom (at *n* = 3000 rpm and *f* = 150 mm/min), while the lowest hole circularity at the bottom occurred at *n* = 3000 rpm and *f* = 50 mm/min using TiN/TiAlN-coated tools. Generally, lower hole circularity is preferred; however, an acceptable value for hole circularity can vary depending on the requirements of the machined part, and thus there is no standard for acceptable hole circularity [57]. Nevertheless, it is possible to compare the circularity results in this study with the results from previous literature shown earlier on drilling Al6061 and other aluminum alloys [21,39,61]. That said, previous literature shows that the circularity ranged as low as 4 μm and as high as 182 μm, which means that the results reported in this study are well within those ranges. Indeed, the results in the current study were similar to those reported by Sreenivasulu et al. on drilling Al6061 alloy [21].

The ANOVA analysis provided in Table 5 shows that both cutting parameters and tool coating had a significant influence on hole circularity at the top. The tool coating had the highest contribution, with 22.48%, followed by the spindle speed and the feed rate, with 10.35% and 4.06%, respectively. The interaction between the spindle speed and the tool coating had a significant impact of 16.71%, which shows that a proper combination of tool coating and spindle speed can reduce the circularity error in holes drilled in Al6061 alloy. For hole circularity at the bottom, only the feed rate and the tool coating had a significant impact, with 6.08% and 19.53%, respectively. This indicates that the cutting parameters (spindle speed and feed rate) had different effects on hole circularity, depending on the measured location throughout the hole depth. A firm conclusion can be reached that better control of spindle speed at the top and better control of the feed rate at the bottom will result in the best hole circularity. The results reported here are in agreement with previous studies, which found that both cutting parameters had a significant influence on the hole circularity, with spindle speed showing a larger contribution [38,62].

### 3.4. Effect of Cutting Parameters and Tool Coatings on Hole Cylindricity and Perpendicularity

**Figure 10:**
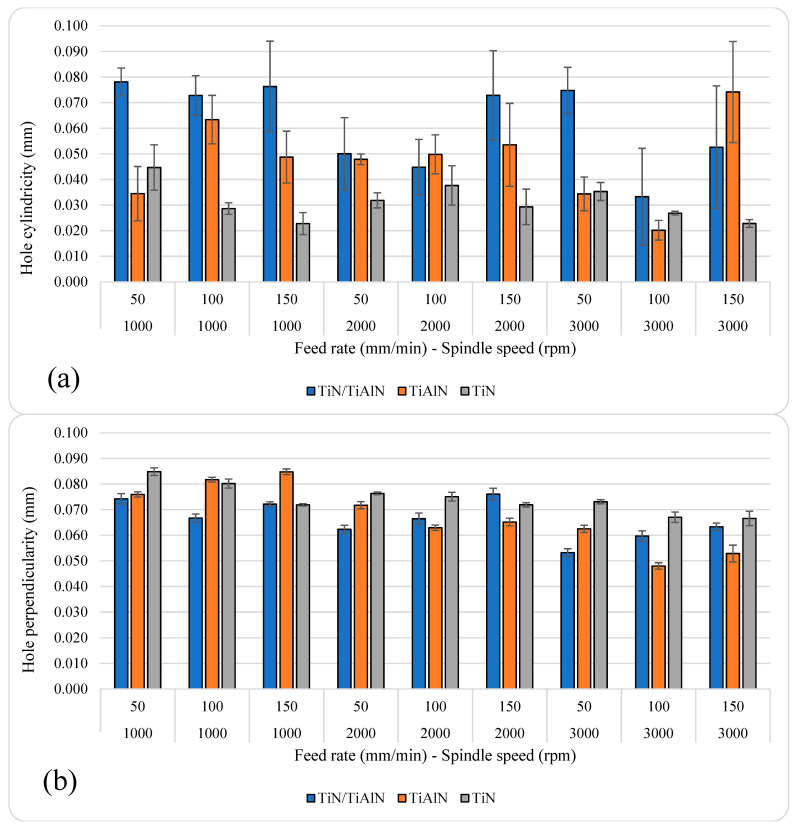
Average hole (**a**) cylindricity and (**b**) perpendicularity.

Figure 10 shows the average hole cylindricity and perpendicularity under different cutting parameters and tool coatings. The average hole cylindricity ranged between 20.17 and 78.10 µm, while the average hole perpendicularity ranged between 48 and 84.83 µm, as shown in Table 8. The highest hole cylindricity and perpendicularity occurred at *n* = 1000 rpm and *f* = 50 mm/min in holes drilled using TiN/TiAlN-coated tools. The lowest hole cylindricity and perpendicularity occurred at *n* = 3000 rpm and *f* = 1000 mm/min in holes drilled using TiAlN-coated tools.

The range of cylindricity error in the current study is relatively low if it is compared to the hole cylindricity error reported in previous studies, which reached up to 260 µm in the aluminum alloy [63]. However, the cylindricity error observed in the current study had a similar range for that when aluminum-silicon nitride is drilled with a conventional method [64]. For TiN, the cylindricity error was reduced by increasing the feed rate at *n* = 1000 and *f* = 3000 rpm. For other coatings, no clear relationship could be found between the cylindricity error and feed rate. For TiN/TiAlN, the cylindricity error was inversely related to spindle speed at a feed rate of *f* = 100 and 150 mm/min. For TiAlN, the relation was inverse at *f* = 100 mm/min and proportional at *f* = 150 mm/min. Figure 10 also shows a reduction in the cylindricity error for TiN coating when increasing the feed rate and spindle speed, but keeping the ratio of the constant as 50/1000, 100/2000, and 150/3000.

Overall, it was observed that holes drilled using TiN-coated tools showed the highest perpendicularity, especially when drilling at medium and high spindle speeds in the study (*n* = 2000 and 3000 rpm). Similarly, holes drilled using TiAlN-coated tools gave lower perpendicularity values compared to those observed in holes drilled using TiN/TiAlN-coated tools when drilling at *n* = 2000 and 3000 rpm. For these two coatings, i.e., TiN/TiAlN and TiAlN, their performance in reducing hole perpendicularity became noticeable when drilling at higher spindle speeds and increasing feed rate. This means that these coatings are more suitable for high-speed machining applications due to the superior performance of their coating characteristics over TiN coating when drilling aluminum alloys. In general, the perpendicularity error was reduced by increasing the spindle speed. For TiN/TiAlN, increasing the feed rate increased the perpendicularity error at *n* = 2000 and 3000 rpm, while the opposite was almost true for TiAlN and TiN at the same speeds. Similar to the cylindricity error, the range of perpendicularity error in the current study was less than that found for aluminum drilled with cryogenic cooling [63], and it was much less than aluminum drilled using abrasive-water-jet machining [65], with the annotation that different federates and spindle speeds were used in the aforementioned studies.

### 3.5. Cutting-Tool Examination

**Figure 11:**
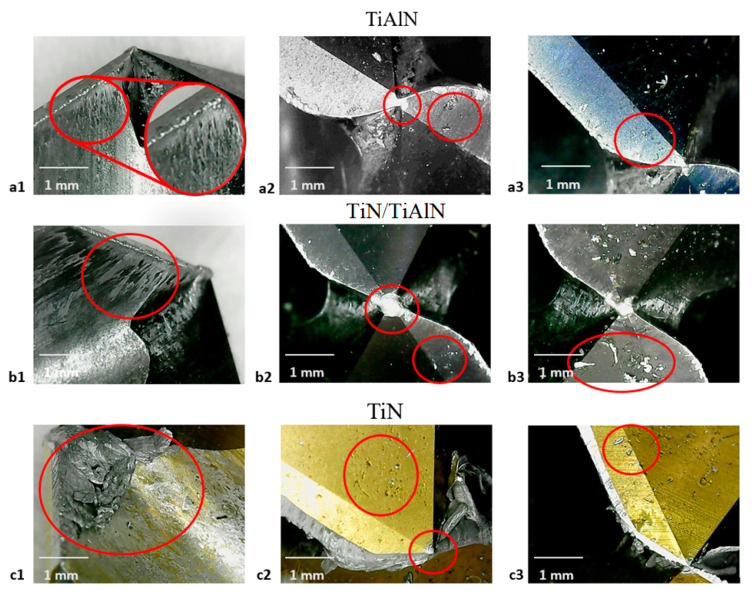
(**a1**,**b1**,**c1**) Built-up edge and aluminum spread adhesion; (**a2**,**b2**,**c2**) surface pitting and aluminum build-up on tool’s tip; (**a3**,**b3**,**c3**) aluminum filling the surface pitting.

The microconstituents found in aluminum alloys have an essential influence on the properties of the machining. Nonabrasive components have a beneficial effect, and insoluble abrasive components can have a harmful impact on surface quality. Insoluble but flexible and nonabrasive components are advantageous, as they help split the chips; these components are deliberately used to formulate high-strength free-cutting alloys for machining in high-speed automated machines. Generally, the softer alloys and, to a lesser degree, some of the stronger alloys are likely to develop a built-up edge on the tool’s cutting surface. This edge consists of aluminum particles that have been welded to the edge of the tool because the heat produced in cutting has melted them. Figure 11 shows the optical microscopic inspection of the tools after drilling. In this figure, the built-up edge can be seen on all tools, but it is relatively more built-up on the TiN, which is in line with results reported by Giasin et al. [12] when drilling fiber metal laminates containing sheets of aluminum alloy. This may be due to the lower thermal stability of TiN under the high temperatures generated due to dry drilling. It can also be seen that some of the aluminum particles adhered to the surface of the tool. These particles were melted and squashed on the tool’s surface due to the high speed. The accumulation and adhesion of aluminum on the tip were observed for all tools. Pitting was also observed for all tools, but there was relatively more on the surface of the TiN tools. These pittings were sometimes filled with alumina particles. Based on the optical examination, the surface and the edge of the TiN tool were most affected by the operating conditions in this study. However, a conclusion on the tool life for each of the coated tools could not be reached due to the limited number of holes drilled in this study.

**Figure 12:**
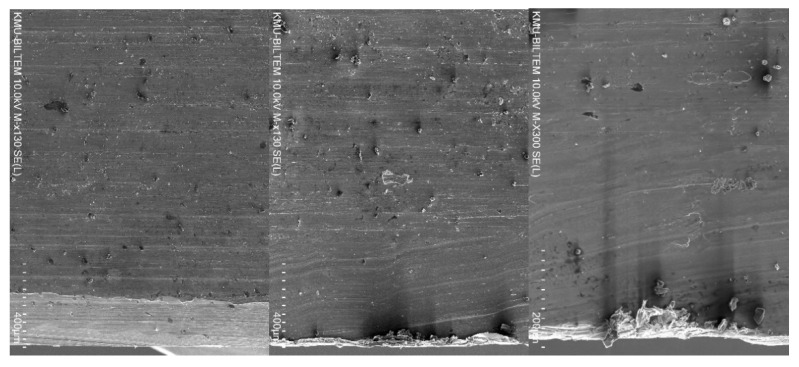
Hole condition at the exit showing edge quality and burr formation for holes drilled at *n* = 3000 rpm and *f* = 50 mm/min (From left to right: using TiN/TiAlN, TiAlN and TiN coating).

The SEM images in Figure 12 revelated that the burr formation and hole edge quality at the exit was the worst when drilling using TiN-coated tools, followed by TiAlN-coated tools. For TiN/TiAlN-coated tools, despite the formation of the white layer at the exit side of the hole, the burr formation and edge quality were superior to the hole drilled using the other two tool coatings. This would indicate that drilling using TiN/TiAlN-coated tools would produce holes with the minimal burr at the exit, thus avoiding the need for any deburring operations, which is a highly desirable outcome in the aerospace industry, where large numbers of holes are drilled in a short period, and deburring them would add extra costs to the overall manufacturing process. At the hole entry, no conclusion could be made regarding which tool coating produced the best edge quality and least burr formation, as all holes gave somewhat similar results.

## 4. Conclusions

In this study, the drilling performance of Al6061 aluminum alloy was analyzed to investigate the hole surface roughness, form, and dimensional tolerances. More specifically, these included hole size and circularity at the upper and lower portions, and cylindricity and perpendicularity. The aim of the study was to analyze the influence of cutting parameters (spindle speed and feed rate), and particularly, three types of cutting-tool coatings (TiN/TiAlN, TiAlN, and TiN) using solid carbide twist drills. This work aims to complement the tool-coating studies reported in the open literature and fill the gap, since the effect of these three types of cutting tool coatings on drilling Al6061 aluminum alloys was not previously investigated in a single study. Moreover, this work specifically aimed to study the effect of tool coatings by using a fixed tool geometry (i.e., same drill size, point angle, helix angles, tool hardness, etc.). The contribution of cutting-tool coatings is considered a critical aspect when machining aluminum alloys to better understand how they influence the quality of machined parts. From the experimental results and statistical analysis, the following results were obtained:

- The surface-roughness metrics *R*_a_ and *R*_z_ in most holes did not exceed 1 µm and 3 µm, respectively. Holes drilled using TiN-coated tools had the highest surface roughness. This was mainly attributed to the lower oxidation temperature and hardness of TiN coating and its high affinity to react with aluminum during the drilling process, as evident in the microscopic images.
- Hole size and circularity at the top were better than those at the bottom regardless of the tool coating or cutting parameters used. TiN/TiAlN-coated tools produced the worst hole size and circularity at the top, especially at low and medium spindle speeds, followed by holes drilled using TiN and TiAlN coatings.
- Under all cutting conditions, the holes produced were always oversized (between 15 and 80 µm). Similarly, hole circularity at the top and bottom ranged between 7 and 60 µm.
- TiN/TiAlN-coated tools produced the worst hole cylindricity, followed by the TiAlN and TiN tools. Hole cylindricity was the worst in TiN/TiAlN-coated tools, especially at low and medium spindle speeds. Holes drilled using TiN-coated tools gave the lowest hole cylindricity among the three coatings.
- TiN coatied tools were more suitable for drilling Al6061 alloy at low cutting parameters, while TiN/TiAlN- and TiAlN-coated tools were more suitable when machining at higher cutting parameters, where the thermal performance of the tool coating became more critical to the quality of machined holes.
- The ANOVA results showed that both the cutting parameters and the cutting-tool coatings had an impact on hole size, circularity, cylindricity, and perpendicularity. The contribution of the cutting-tool coating was more significant at the hole entry than at the exit. The spindle speed had a major effect on hole perpendicularity compared to feed rate and type of tool coating.

## Figures and Tables

**Table 2:** Al6061-T651 alloy material properties Adapted with permission from ref. [36]. Copyright 2017 Elsevier, and component element properties Adapted with permission from ref. [37]. Copyright 2012 Elsevier.

Properties	Metric	Element	Percentage
Density (g/cc)	2.7	Aluminum (Al)	98
Ultimate Tensile Strength (MPa)	310	Chromium (Cr)	0.04–0.35
Tensile Yield Strength (MPa)	276	Copper (Cu)	0.15–0.4
Modulus of Elasticity (GPa)	68.9	Iron (Fe)	0.7
Bearing Yield Strength (MPa)	386	Magnesium (Mg)	0.8–1.2
Poisson’s Ratio	0.33	Manganese (Mn)	0.15
Fatigue Strength (MPa)	96.5	Silicon (Si)	0.4–0.8
Fracture Toughness (MPa-m½)	29	Titanium (Ti)	0.15
Machinability (%)	50	Zinc (Zn)	0.25
Shear Modulus (GPa)	26		
Specific Heat Capacity (J/g-°C)	0.896		
Melting Point (°C)	582–652		
Hardness (HV)	107		

**Table 3:** Details of the cutting-tool materials and coatings Adapted with permission from ref. [12]. Copyright 2019 Elsevier.

Description	Tool A	Tool B	Tool C
Tool material	Solid carbide	Micrograin carbide	Solid carbide
Drill diameter (mm)	10	10	10
Helix angle (°)	30	30	30
Point angle (°)	140	140	140
Tolerance	M7	M7	M7
Coating	Firex coating (TiN/TiAlN)	TiAlN	TiN
Flute length	47	47	43
Overall length	89	89	89
Color	Red violet	Black violet	Gold
Coating thickness (μm)	1.5–5	1.5–4	1.5–3
Layer structure	Multilayer	Monolayer	Monolayer
Nano hardness (HV 0.05)	3000–3300	3300	2400
Friction coefficient	0.5	0.5–0.55	0.4–0.5
Oxidation resistance (°C)	930	700–800	593
Manufacturer	GUHRING (Germany)	OSG (Japan)	GUHRING (Germany)

**Table 4:** Details of cutting parameters and tool coatings used in the study.

Factor	Level 1	Level 2	Level 3
Spindle speed (rpm)	1000	2000	3000
Feed rate (mm/min)	50	100	150
Coating	TiN/TiAlN	TiAlN	TiN

**Table 5:** ANOVA analysis to show the percentage contribution of spindle speed, feed rate, and coating type for the cutting tool on the investigated hole-quality parameters.

Parameter	*R*_a_	*R*_z_	HST	HSB	HCT	HCB	HC	HP
Model	50.79%	50.48%	91.91%	86.34%	64.50%	60.94%	73.62%	97.00%
Blocks	0.34%	0.42%	0.32%	1.80%	0.70%	1.27%	0.83%	0.09%
**Linear**	36.81%	35.95%	42.73%	23.94%	36.88%	26.04%	41.30%	66.75%
Spindle speed	27.47%	26.02%	16.89%	3.75%	10.35%	0.43%	4.14%	50.61%
Feed rate	4.11%	4.88%	1.95%	3.39%	4.06%	6.08%	2.75%	1.68%
Coating	5.23%	5.05%	23.90%	16.80%	22.48%	19.53%	34.41%	14.46%
**2-Way Interactions**	6.03%	6.35%	35.11%	47.32%	21.73%	15.64%	20.04%	19.93%
Spindle speed*Feed rate	3.00%	3.13%	13.63%	11.80%	0.15%	5.89%	6.84%	0.66%
Spindle speed*Coating	2.76%	3.13%	19.24%	26.99%	16.71%	6.63%	4.17%	10.79%
Feed rate*Coating	0.27%	0.09%	2.24%	8.52%	4.87%	3.11%	9.04%	8.48%
**3-Way Interactions**	7.61%	7.76%	13.74%	13.27%	5.19%	18.00%	11.45%	10.23%
Spindle speed*Feed rate*Coating	7.61%	7.76%	13.74%	13.27%	5.19%	18.00%	11.45%	10.23%
Error	49.21%	49.52%	8.09%	13.66%	35.50%	39.06%	26.38%	3.00%
Total	100.00%	100.00%	100.00%	100.00%	100.00%	100.00%	100.00%	100.00%

Note: HST: hole size (top); HSB: hole size (bottom); HCT: hole circularity error (top); HCT: hole circularity error (bottom); HC: hole cylindricity error; HP: hole perpendicularity error.

**Table 6:** Range of hole-size deviation for each tool coating.

Coating	Top (µm)	Bottom (µm)
TiN/TiAlN	24.23–79.9	20.6–48.13
TiAlN	17.83–34.87	19.87–29.17
TiN	25.13–51.53	15.47–32.7

**Table 7:** Range of hole-circularity errors for each tool coating.

Coating	Top (µm)	Bottom (µm)
TiN/TiAlN	18.33–59.63	7.03–15.13
TiAlN	6.63–42.2	8.93–15.6
TiN	12–26.4	9.3–19.7

**Table 8:** Range of hole cylindricity and perpendicularity for each tool coating.

Coating	Cylindricity (µm)	Perpendicularity (µm)
TiN/TiAlN	33.27–78.10	53.23–76.03
TiAlN	20.17–74.17	48–84.77
TiN	22.77–44.67	66.57–84.83

## Data Availability

The data presented in this study are available on request from the corresponding author.

## References

[1] Schmidt A., Siebeck S., Götze U., Wagner G., Nestler D. (2018). Particle-Reinforced Aluminum Matrix Composites (AMCs)—Selected Results of an Integrated Technology, User, and Market Analysis and Forecast. Metals.

[2] Davis J.R. (1999). Corrosion of Aluminum and Aluminum Alloys.

[3] Joseph R., Davis J. (1993). Aluminium and Aluminium Alloys. Asm Int. Mater..

[4] Kermanidis A.T. (2020). Aircraft Aluminum Alloys: Applications and Future Trends. Revolutionizing Aircraft Materials and Processes.

[5] Gupta M.K., Mia M., Singh G., Pimenov D.Y., Sarikaya M., Sharma V.S. (2019). Hybrid cooling-lubrication strategies to improve surface topography and tool wear in sustainable turning of Al 7075-T6 alloy. Int. J. Adv. Manuf. Technol..

[6] Siwawut S., Saikaew C., Wisitsoraat A., Surinphong S. (2018). Cutting performances and wear characteristics of WC inserts coated with TiAlSiN and CrTiAlSiN by filtered cathodic arc in dry face milling of cast iron. Int. J. Adv. Manuf. Technol..

[7] Myasnikov Y.I., Pimenov D.Y. (2016). Fast drilling of small-diameter holes by core flat drills. Russ. Eng. Res..

[8] Myasnikov Y.I., Pimenov D.Y. (2016). High-speed drilling of small-diameter holes by core flat drills. Russ. Eng. Res..

[9] Pan J., Ni J., He L., Cui Z., Feng K. (2020). Influence of micro-structured milling cutter on the milling load and surface roughness of 6061 aluminum alloy. Int. J. Adv. Manuf. Technol..

[10] Sarikaya M., Gupta M.K., Tomaz I., Danish M., Mia M., Rubaiee S., Jamil M., Pimenov D.Y., Khanna N. (2020). Cooling techniques to improve the machinability and sustainability of light-weight alloys: A state-of-the-art review. J. Manuf. Process..

[11] Okokpujie I.P., Ikumapayi O.M., Okonkwo U.C., Salawu E.Y., Afolalu S.A., Dirisu J.O., Nwoke O.N., Ajayi O.O. (2017). Experimental and mathematical modeling for prediction of tool wear on the machining of aluminium 6061 alloy by high speed steel tools. Open Eng..

[12] Giasin K., Gorey G., Byrne C., Sinke J., Brousseau E. (2019). Effect of machining parameters and cutting tool coating on hole quality in dry drilling of fibre metal laminates. Compos. Struct..

[13] Uddin M., Basak A., Pramanik A., Singh S., Krolczyk G.M., Prakash C. (2018). Evaluating Hole Quality in Drilling of Al 6061 Alloys. Materials.

[14] Boswell B., Islam M.N., Davies I.J., Pramanik A. (2017). Effect of machining parameters on the surface finish of a metal matrix composite under dry cutting conditions. Proc. Inst. Mech. Eng. Part. B J. Eng. Manuf..

[15] Kumar C.R., JaiGanesh V., Malarvannan R.R.R. (2019). Optimization of drilling parameters in hybrid (Al6061/SiC/B 4 C/talc) composites by grey relational analysis. J. Braz. Soc. Mech. Sci. Eng..

[16] Karabulut Ş., Karakoç H., Çıtak R. (2016). Influence of B4C particle reinforcement on mechanical and machining properties of Al6061/B4C composites. Compos. Part. B Eng..

[17] Motorcu A.R., Kuş A., Durgun İ. (2014). The evaluation of the effects of control factors on surface roughness in the drilling of Waspaloy superalloy. Measurement.

[18] Choudhary R., Singh G. (2017). Effect of machining parametrs on metal removal rate during electric discharge drilling of Al 6061 with hollow Cu electrode. Int. J. Des. Manuf. Technol..

[19] Sreenivasulu R., Rao C. (2012). Application of grey relational analysis for surface roughness and roundness error in drilling of Al 6061 alloy. Int. J. Lean Think..

[20] Ravikumar H., Arun P., Thileepan S. (2015). Analysis in Drilling of Al6061/20% SiCp Composites Using Grey Taguchi Based TOPSIS (GT-TOPSIS). Int. J. Chem.Tech. Res. (IJCRGG).

[21] Sreenivasulu R. (2015). Optimization of burr size, surface roughness and circularity deviation during drilling of Al 6061 using Taguchi design method and artificial neural network. Indep. J. Manag. Prod..

[22] Narahari P., Pai B.C., Pillai R.M. (1999). Some aspects of machining cast Al-SiCp composites with conventional high speed steel and tungsten carbide tools. J. Mater. Eng. Perform..

[23] Faverjon P., Rech J., Leroy R. (2013). Influence of Minimum Quantity Lubrication on Friction Coefficient and Work-Material Adhesion During Machining of Cast Aluminum With Various Cutting Tool Substrates Made of Polycrystalline Diamond, High Speed Steel, and Carbides. J. Tribol..

[24] Roy P., Sarangi S.K., Ghosh A., Chattopadhyay A.K. (2009). Machinability study of pure aluminium and Al–12% Si alloys against uncoated and coated carbide inserts. Int. J. Refract. Met. Hard Mater..

[25] Thakre A.A., Soni S. (2016). Modeling of burr size in drilling of aluminum silicon carbide composites using response surface methodology. Eng. Sci. Technol. Int. J..

[26] Islam M.N., Boswell B. Effect of cooling methods on hole quality in drilling of aluminium 6061-6T. Proceedings of the IOP Conference Series: Materials Science and Engineering.

[27] Ranjan J., Patra K., Szalay T., Mia M., Gupta M.K., Song Q., Krolczyk G., Chudy R., Pashnyov V.A., Pimenov D.Y. (2020). Artificial Intelligence-Based Hole Quality Prediction in Micro-Drilling Using Multiple Sensors. Sensors.

[28] Myasnikov Y.I., Pimenov D.Y. (2016). Optimizing the high-speed drilling of small-diameter holes by core flat drills. Russ. Eng. Res..

[29] Sun D., Lemoine P., Keys D., Doyle P., Malinov S., Zhao Q., Qin X., Jin Y. (2018). Hole-making processes and their impacts on the microstructure and fatigue response of aircraft alloys. Int. J. Adv. Manuf. Technol..

[30] Gu W., Xu H., Liu J., Yue Z. (2009). Effect of drilling process on fatigue life of open holes. Tsinghua Sci. Technol..

[31] Blau P., Martin R., Riester L. (1996). A Comparison of Several Surface Finish Measurement Methods as Applied to Ground Ceramic and Metal Surfaces.

[32] Kivak T., Habali K., Şeker U. (2012). The effect of cutting paramaters on the hole quality and tool wear during the drilling of Inconel 718. Gazi Univ. J. Sci..

[33] Sheth S., George P. (2016). Experimental investigation, prediction and optimization of cylindricity and perpendicularity during drilling of WCB material using grey relational analysis. Precis. Eng..

[34] Giasin K. (2018). The effect of drilling parameters, cooling technology, and fiber orientation on hole perpendicularity error in fiber metal laminates. Int. J. Adv. Manuf. Technol..

[35] Bartkowiak T., Brown C.A. (2019). Multiscale 3D curvature analysis of processed surface textures of aluminum alloy 6061 T6. Materials.

[36] Pitchayyapillai G., Seenikannan P., Balasundar P., Narayanasamy P. (2017). Effect of nano-silver on microstructure, mechanical and tribological properties of cast 6061 aluminum alloy. Trans. Nonferrous Met. Soc. China.

[37] Bang H., Bang H., Jeon G., Oh I., Ro C. (2012). Gas tungsten arc welding assisted hybrid friction stir welding of dissimilar materials Al6061-T6 aluminum alloy and STS304 stainless steel. Mater. Des..

[38] Giasin K., Ayvar-Soberanis S., Hodzic A. (2015). An experimental study on drilling of unidirectional GLARE fibre metal laminates. Compos. Struct..

[39] Giasin K., Hodzic A., Phadnis V., Ayvar-Soberanis S. (2016). Assessment of cutting forces and hole quality in drilling Al2024 aluminium alloy: Experimental and finite element study. Int. J. Adv. Manuf. Technol..

[40] Islam M., Boswell B. Effect of cooling methods on cutting temperature, cutting force and hole quality in drilling of three ferrous alloys. Proceedings of the International Conference on Mechanical and Manufacturing Engineering (ICME2018).

[41] Goindi G.S., Sarkar P. (2017). Dry machining: A step towards sustainable machining–Challenges and future directions. J. Clean. Prod..

[42] Yıldırım Ç.V., Kıvak T., Erzincanlı F. (2019). Influence of Different Cooling Methods on Tool Life, Wear Mechanisms and Surface Roughness in the Milling of Nickel-Based Waspaloy with WC Tools. Arab. J. Sci. Eng..

[43] Gadelmawla E.S., Koura M.M., Maksoud T.M.A., Elewa I.M., Soliman H.H. (2002). Roughness parameters. J. Mater. Process. Technol..

[44] Aamir M., Tolouei-Rad M., Giasin K., Vafadar A. (2020). Machinability of Al2024, Al6061, and Al5083 alloys using multi-hole simultaneous drilling approach. J. Mater. Res. Technol..

[45] Kurt M., Kaynak Y., Bagci E. (2008). Evaluation of drilled hole quality in Al 2024 alloy. Int. J. Adv. Manuf. Technol..

[46] Bresseler B., El-Wardany T., Elbestawi M. Material side flow in high speed finish boring of case hardened steel. Proceedings of the 1st French and German Conference on High Speed Machining.

[47] Kishawy H., Elbestawi M.A. (1999). Effects of process parameters on material side flow during hard turning. Int. J. Mach. Tools Manuf..

[48] Whitehouse D.J. (2010). Handbook of Surface and Nanometrology.

[49] Giasin K. (2017). Machining Fibre Metal Laminates and Al2024-T3 Aluminium Alloy. Ph.D. Thesis.

[50] Coromant S. (2010). Machining carbon fibre materials. Sandvik Coromant User’s Guide-Composite Solutions.

[51] Nouari M., List G., Girot F., Coupard D. (2003). Experimental analysis and optimisation of tool wear in dry machining of aluminium alloys. Wear.

[52] Kurt M., Bagci E., Kaynak Y. (2009). Application of Taguchi methods in the optimization of cutting parameters for surface finish and hole diameter accuracy in dry drilling processes. Int. J. Adv. Manuf. Technol..

[53] Shareef I., Natarajan M., Ajayi O.O. Dry machinability of aluminum alloys. Proceedings of the World Tribology Congress III.

[54] Abdelhafeez A.M., Soo S.L., Aspinwall D.K., Dowson A., Arnold D. (2015). Burr Formation and Hole Quality when Drilling Titanium and Aluminium Alloys. Procedia CIRP.

[55] Markopoulos A.P., Papazoglou E.-L., Karmiris-Obratański P. (2020). Experimental study on the influence of machining conditions on the quality of electrical discharge machined surfaces of aluminum alloy Al5052. Machines.

[56] GD&T GD&T: Perpendicularity. https://www.gdandtbasics.com/perpendicularity/.

[57] Giasin K., Hawxwell J., Sinke J., Dhakal H., Köklü U., Brousseau E. (2020). The effect of cutting tool coating on the form and dimensional errors of machined holes in GLARE® fibre metal laminates. Int. J. Adv. Manuf. Technol..

[58] Giasin K., Ayvar-Soberanis S., French T., Phadnis V. (2016). 3D Finite Element Modelling of Cutting Forces in Drilling Fibre Metal Laminates and Experimental Hole Quality Analysis. Appl. Compos. Mater..

[59] AlSi I. (2017). Effect of cutting parameters on the drilling of AlSi7 metallic foams. Mater. Tehnol..

[60] Hayajneh M.T. (2001). Hole quality in deep hole drilLING. Mater. Manuf. Process..

[61] Zitoune R., Krishnaraj V., Collombet F. (2010). Study of drilling of composite material and aluminium stack. Compos. Struct..

[62] Giasin K., Ayvar-Soberanis S. (2017). An Investigation of burrs, chip formation, hole size, circularity and delamination during drilling operation of GLARE using ANOVA. Compos. Struct..

[63] Govindaraju N., Shakeel A.L., Pradeepkumar M. (2014). Experimental investigations on cryogenic cooling in drilling of aluminium alloy. Applied Mechanics and Materials.

[64] Gowda B.M.U., Ravindra H.V., Jain S.P., Raj M.N., Prakesh G.V.N., Ugrasen G. (2014). Comparative Study of Surface Roughness and Cylindricity of Aluminium Silicon Nitride Material Using MRA GMDH & Pattern Recognition Technique in Drilling. Procedia Mater. Sci..

[65] Herghelegiu E., Radu M.C., Schnakovszky C., Tampu C.N. Considerations on material thickness influence on the AWJ processing quality of an aluminium alloy. Proceedings of the MATEC Web of Conferences.

